# Early discontinuation of long-acting reversible contraceptives at four government hospitals, Addis Ababa, Ethiopia

**DOI:** 10.1186/s40834-023-00238-8

**Published:** 2023-07-21

**Authors:** Adane Sisay, Abel Teshome, Hailemichael Bizuneh, Sarah D.Compton

**Affiliations:** 1 Department of Obstetrics and Gynecology, Saint Paul’s Hospital Millennium Medical College, St. Paul’s Hospital Millennium Medical College, Addis Ababa, Ethiopia; 2 School of Public Health, Department of Epidemiology, Saint Paul’s Hospital Millennium Medical College, Addis Ababa, Ethiopia; 3 Department of Obstetrics and Gynecology, University of Michigan Medical School, Ann Arbor, MI USA

**Keywords:** Early Discontinuation, Long-acting reversible contraception, Family planning

## Abstract

**Objective:**

Given high unmet need for contraception in Ethiopia, this study aimed to determine prevalence and factors associated with early discontinuation of long-acting reversible contraceptives (LARC).

**Methods:**

This institution-based cross-sectional study was conducted with 389 participants using exit interviews with clients presenting for removal of LARC at the family planning clinic of four government hospitals in Addis Ababa.

SPSS version 26 was used for analysis. Descriptive statistics, bivariate, and multivariate logistic regression were computed.

**Result:**

Among the 389 clients, 236 (60.7%) discontinued early. In multivariate regression, lack of pre-insertion counseling on side effects (AOR = 3.5, *p* = 0. 000; 95% C.I = 1.8—6.8), presence of side effects (AOR = 1.9, *p* = 0. 017; 95% C.I = 1. 1- 3.4), history of abortion (AOR = 3.5, *p* = 0. 001; 95% C.I = 1. 6- 7.4); and no prior contraception use (AOR = 2.9, *p* = 0. 000; 95% C.I = 1. 6- 5.3) were positively associated with early discontinuation. Whereas insertion outside of Saint Paul’s Hospital Millennium Medical College (AOR = 0.4, *p* = 0. 000; 95% C.I = 0. 2- 0.6), and influence on choice of contraceptives by others (AOR = 0.2, *p* = 0. 000; 95% C.I = 0. 2- 0.4) were negatively associated with early discontinuation.

**Conclusion:**

Early discontinuation of LARC was high among study participants. Counseling about possible side effects and giving women the opportunity to decide their own choice of contraception might help in reducing early discontinuation.

## Synopsis

Counseling about side effects and allowing women to decide on their contraception might help reduce the high rate of early discontinuation of long-acting reversible contraception

## Introduction

Long-acting reversible contraceptive (LARC) methods, which include intrauterine contraceptive devices (IUD) and implants, are widely available in Ethiopia [1].

LARC methods are among the longest-acting and effective ways to prevent pregnancy; copper IUDs are effective for up to 10 years [2], while the hormonal IUD, LNG-20, is effective for up to 5 years [3]. Implants (Implanon and Jadelle) are also long-acting and extremely effective in preventing pregnancy, and are effective for 3 and 5 years respectively. All of these methods have a less than one percent clinical failure rate [1, 2]. As these devices do not require users to do anything once they are initiated, the “perfect use” failure rates are also recorded in actual use [4]. LARC methods may be a good option for couples who want to delay pregnancy for a longer period or limit any future pregnancies but who do not want a permanent method [5].

The public health benefits of family planning are clear; family planning could help prevent 70% of maternal and more than 57% of under-five deaths by prolonging birth intervals and avoiding unwanted pregnancy [6]. Further, it encourages women to engage in the workforce, reduces poverty, and enhances their involvement in political and economic decision making [7]. The American College of Obstetricians and Gynecologists has recommended LARC methods as first-line contraceptive options for both adults and adolescents [8], since, compared with other reversible contraceptive methods, they are compatible with breastfeeding, do not require ongoing effort, are independent of coitus, and are generally safe [9, 10]. In addition, after the device is removed, the return of fertility is rapid and insertion and removal are easy and can be done by a range of medical professionals [3].

While IUDs are used by almost 12 percent of contraceptive users in the United States and 40 percent in China, [5], only 8% of married women in Ethiopia use implants and 2% IUDs [11].

While the devices are expensive, many countries provide the service free of cost in order to remove financial barriers which could prevent women from accessing these highly effective devices [8]. In a low-income countries like Ethiopia, approximately 225 million people had an unmet need for modern contraception [12]. The percentage of Ethiopian women of reproductive age who still lack access to family planning declined from 39.6% in 2005 to 23.6% in 2016 [13].

The Ethiopian family planning program, like in other developing countries, is experiencing the leaking bucket phenomenon [14], where more people are discontinuing contraception than start it. Apart from low utilization, premature removal of LARC has been documented to occur in 16 to 46.5% of new episodes [11, 15]. Age at the time of discontinuation, family size, fertility preferences, the prior use of a method, the contraceptive method chosen, and prior experience with the method have been shown to be predictors of discontinuation [12]. Side effects or health concerns were the reason given for four of ten episodes of LARC discontinuations [7].

All previous studies of LARC discontinuation in Ethiopia used a community-based approach with varying study periods. hey have reported a wide range of discontinuation rates of LARC [1, 16, 17]. Therefore, this study was conducted in four large hospitals of Addis Ababa and aimed to determine the prevalence of early discontinuation of LARC and associated factors for early discontinuation among a facilty-based population.

## Materials and methods

This cross-sectional study was conducted at the family planning clinics of four hospitals in Addis Ababa; Saint Paul’s Hospital Millennium Medical College, Abebech Gobena Maternal and Child health hospital, Ras Desta Damtew Memorial hospital and Minilik II hospital. The data collection was conducted over a 1-year period from June 2021 to June 2022. Ethical approval of and clearance for the study was obtained from the Ethical Review Board of Saint Paul’s Hospital Millennium Medical College before the start of data collection. Informed consent was obtained from all women who were interviewed.

The data were collected Monday to Friday from 8:00 AM to 5:00 PM by BSc Midwives through exit interviews at family planning clinics of those four hospitals. On average, 1 to 5 clients/day were served for LARC removal at each hospital. Data were collected by total of 10 BSC midwives working in those Governmental hospitals. The LARC removal service at each hospital was provided by midwives, medical interns, and residents. All reproductive aged women 15–49 years who came for LARC removal were included. Those women over 49 years of age and at their device’s due date were excluded. All eligible participants were invited for interviews, but 10 of them declined interview and all of 10 replaced by other eligible volunteers to achieve total sample size.

The sample size was calculated by using the Kish formula, *n* = Z^2^P (1-P)/ W^2^, where Z is Z- score, P is percentage, and W is width (precision). A previous study showed that 21.5% of women had early LARC removal [18]. To reduce sampling error, we used a design effect of 1.5, a precision of 0.05, and a 95% confidence interval. With the addition of 10% to account for non-response, a total sample size of 389 was calculated.

The questionnaire was developed through literature and expert review [15], translated to Amharic (the local language), and validated by back-translation to English. Data quality was assured by using this validated questionnaire, by training the data collectors, and by pre-testing the data collection tool on 5% of the study participants. Data collection started without modification on the pretest questionnaire. WHO recommends optimal pregnancy spacing as 24 months [6, 8]. Based on this, the dependent variable was dichotomized as early removal of LARC before 24 months yes/no. After the completeness and coding of the questionnaires was checked, quantitative data were analyzed by using SPSS version 26 for Windows (IBM, Armonk, NY, USA). Data were reported as number (percentage) and a p-value of less than 0.05 was taken to be significant.

Logistic regression was used to evaluate association with early discontinuation of LARC. The independent variables, which were informed by reviewing previous studies [15] and modified based on factors the authors felt important were age, parity, monthly income, educational status, religion, maternal occupation, place of insertion, faculty of insertion, maternal involvement in LARC selection, partner's occupation, family size, previous contraceptive use, pre-insertion counseling, side effects, and reason for discontinuation of LARC.

## Results

A total of 389 women completed the questionnaire. Participants were between 16 and 45 years with mean (± SD) age of 27.6 ± 5.6 years. More than three quarters, 347 (89.2%), of respondents were married, and 243 (62.5%) were Orthodox.

Less than one-third were housewives (110 (28.3%)) and only 39 (10%) had no formal education. Only 25 (6.4%) had estimated monthly income of below the poverty line (< 1,566 ETB). See Table 1 for complete demographic information about the sample.

**Table 1:** Socio-demographic status of respondents at Addis Ababa Government hospitals, Addis Ababa Ethiopia, 2022 (*n* = 389)

Variables	Frequency	Percentage
**Age (yrs)**		
< 18	5	1.3
19–25	136	35
26–35	213	54.7
> 35	35	9
Age mean ± SD, (Range) yrs	27.6 ± 5.6 (16–45)	
**Marital status**		
Married	347	89.2
Others (single, separated, divorced, widowed)	42	10.8
**Religion**		
Orthodox	243	62.4
Muslim	73	18.8
Others (Protestant, Catholic)	73	18.8
**Educational status**		
No formal education	39	10
Primary	81	20.8
Secondary	131	33.7
College and above	138	35.5
**Monthly income (ETB)**		
Below poverty line (< 1,566)	25	6.4
Above poverty line (> 1,566)	364	93.6

Below poverty line: Daily income < 1$/day, above poverty line: daily income > 1$/day

Three hundred thirty-nine (87.2%) of the respondents had given birth at least one time and 235 (60.4%) had 1–2 alive children. Almost one-third (114 (29.3%)) had a history of abortion, and out of the 235 (60.8%) who had desire for future pregnancy, 192 (49.3%) of them wanted to become pregnant within 2 years (Table 2).

**Table 2:** Maternal reproductive characteristics of respondents at Addis Ababa Government hospitals, Addis Ababa, Ethiopia, 2022 (*n* = 389)

Variables	Frequency	Percentage
**Parity**		
Nulliparous	50	12.8
Parous	339	87.2
**Number of living children**		
0	67	17.2
1–2	235	60.4
3–4	83	21.3
≥ 5	4	1
**History of abortion**		
Yes	114	29.3
No	275	70.7
**Timing of Pregnancy (*n* = 235)**		
Within 2yrs	192	49.3
After 2 yrs	43	11.1

Overall, 236 (60.7%) were determined to be early (< 24 months) discontinuations. Participants were mainly discontinuing the Implanon/Nexplanon implants (275; 70.7%). More than half of the discontinuation of Implanon/Nexplanon were early (187; 68%), while less than half of the IUCD users (32; 41.6%) and Jadelle (17; 45.9%) users were discontinuing early. The mean (± SD) months of use was 22.9 ± 13 months.

Two hundred thirty-nine (61.4%) participants had used modern contraceptives before their currently discontinued LARC, including 109 (45.6%) who had used injectable contraceptives, and 66 (22.7%) Implanon. Of all participants, 295 (75.8%) had their LARC insertion at government hospitals while 64 (16.4%) received it from health centers.

A large majority (368; 94.6%) reported they were counseled on the benefits of LARC prior to insertion, and 245 (63%) were counseled on potential side effects. Further, 227 (58.4%) had chosen LARC on their own while 132 (33.9%) reported LARC was chosen by their health professional. The commonest reasons for choosing the method were fewer side effects (116; 29.8%) and long protection (113; 29%).

Among the reasons for discontinuation of LARC, side effects contributed to 154 (39.6%). The commonest side effect reported was menstrual abnormality (94; 24.2%) for implants, and pain (24; 6.2%) for IUCD (Table 3).

**Table 3:** Contraception related information of respondents at Addis Ababa government hospitals, Addis Ababa, Ethiopia, 2022 (*n* = 389)

Variables	Frequency (N)	Percentage (%)
**Type of LARC discontinued**		
Implanon	275	70.7
IUCD	77	19.8
Jadelle	37	9.5
LARC discontinuation		
Early discontinuation (< 24 months)	236	60.7
Late discontinuation (> 24 months)	153	39.3
Mean ± SD, (range in months)	22.9 ± 13 (3–85)	
**Facility of insertion**		
Government hospital	295	75.8
Health centers	64	16.5
Others ^a^	30	7.7
**Institution of insertion**		
SPHMMC	203	52.2
Other facilities	186	47.8
**Presence of side effects**		
Yes	256	65.8
No	133	34.2
**Counseling on benefit of LARC**		
Yes	368	94.6
No	21	5.4
**Counseling on side effects of LARC**		
Yes	253	65.7
No	132	34.3
**Reason for LARC removal**		
Due to side effects	154	39.6
Need of pregnancy	173	44.5
Others ^b^	62	15.9
**Side effects that resulted Removal (*n* = 160)**		
Menstrual abnormality	94	58.7
Pain	24	15
Weight gain	20	12.5
Others	22	13.7
**Choice of LARC**		
Her own choice	227	58.4
Influence from others ^d^	162	41.6

Other facilities = Health centers, private centers, health posts, Government hospitals other than SPHMMC

^a^ Health post, Private centers

^b^ Divorce, separated, widowed, husband abroad, peer pressure

^c^ Generalized weakness, mood change, vaginal discharge

^d^ Choice by health professional, husband, neighbors and friends

Age, maternal education, parity, number of living children, history of abortion, lack of counseling on side effects, place of insertion, previous contraceptive use, involvement in selection of LARC, and presence of side effects are associated with early discontinuation of LARC in bivariate analysis. (Tables 4 and 5).

**Table 4:** Results of bivariate logistic regression among respondents at Addis Ababa government hospitals, Addis Ababa, Ethiopia, 2022 (*n* = 389)

Variables	Discontinuation		Crude OR (95% CI)	*P*- value	*P*- value
	**Early [**n (%)]	**Late** [n (%)]			
**Choice of LARC**					
Her own choice	93 (41)	134 (59)	4.5 (2.4,8.4)	0.000	0.000
Influence from others	143 (88.3)	19 (11.7)	1		
**Counseling on side effects of LARC**					
Yes	109 (44.5)	136 (54.5)	1		
No	127 (88.2)	17 (11.8)	0.3 (0.1,0.5)	0.000	0.000
**Presence of side effects**					
Yes	173 (67.6)	83 (32.4)	2 (1.2,3.6)	0.012	0.025
No	63 (47.4)	70 (52.6)	1		
**History of contraceptive use before LARC**					
Yes	112 (46.9)	127 (53.1)	1		
No	124 (82.7)	26 (17.3)	0.4 (0.2,0.7)	0.001	0.002
**History of abortion**					
Yes	101 (88.6)	13 (11.4)	3.3 (1.6,7.0)	0.002	0,002
No	135 (49)	140 (51)	1		
**Parity**					
Nulliparous	41 (82)	9 (18)	0.5 (0.2,1.2)	0.126	0.021
Multiparous	195 (57.5)	144 (42.5)			
**Facility of insertion**					
SPHMMC	86 (42.4)	117 (57.6)	0.4 (0.2,0.7)	0.001	0.008
Other facilities ^a^	150 (80.6)	36 (19.4)	1		

^a^ Health centers, private centers, health posts, Government hospitals other than SPHMMC

**Table 5:** Results of multivariate regression among respondents at Addis Ababa government hospitals, Addis Ababa, Ethiopia, 2022 (*n* = 389)

Variables	AOR 95% CI		*P*- value	*P*- value
**Choice of LARC**				
Her own choice	0.2	0.1–0.4	0.000	0.000
Influence from others	1	1		
**Counseling on side effects of LARC**				
Yes	1	1		
No	3.5	1.8–6.8	0.000	0.000
**Presence of side effects**				
Yes	1.9	1.1–3.4	0.017	0.025
No	1	1		
**History of contraceptive use before LARC**				
Yes	1			
No	2.9	1.6–5.3	0.000	0.002
**History of abortion**				
Yes	3.5	1.6–7.4	0.001	0,002
No	1	1		
**Facility of insertion**				
SPHMMC	0.4	0.2–0.6	0.000	0.008
Other facilities ^a^	1	1		

^a^ Health centers, private centers, health posts, Government hospitals other than SPHMMC

In multivariate logistic regression analysis, women who chose LARC had an 80% reduction in early discontinuation compared with insertion influenced by others (AOR = 0.2, *p* = 0. 000; 95% C.I = 0. 1- 0.4), while those who were not counseled about the side effects of LARC before insertion were 3.5 times more likely to discontinue early (AOR = 3.5, *p* = 0. 000; 95% C.I = 1. 8- 6. 8). Early discontinuation is 1.9 times more likely for women who report side effects of LARC compared with those who report they have no side effects AOR = 1.9, *p* = 0.017; 95% C.I = 1. 1- 3.4).

Women were 2.9 times more likely to discontinue early if they had no history of contraceptive use compared to those who had utilized modern contraception before LARC (AOR = 2.9, *p* = 0. 000; 95% C.I = 1. 6- 5.3) and had a 3.5 times increased likelihood if they have history of abortion (AOR = 3.5, *p* = 0. 001; 95% C.I = 1. 6- 7.4).

Early discontinuation was decreased by 60% for those who has LARC insertion at SPHHMC compared with other health facility insertion (AOR = 0.4, *p* = 0. 008; 95% C.I = 0. 2- 0.6). (Table 5).

## Discussion

This study was undertaken to determine the prevalence and associated factors of early discontinuation of long-acting reversible contraception. The key findings of the study are that the overall early discontinuation of LARC was 60.7%, with a mean length of use (± SD) 22.9 ± 13 months. The likelihood of early discontinuation was found to be high among those who reported experiencing side effects. Women who were not counseled about the side effects of contraception, women with history of abortion, and women who had not used modern contraception before this LARC method had a higher likelihood of early discontinuation. Insertion at SPHMMC reduced the likelihood of early discontinuation.

Our rate of early discontinuation is similar to a study conducted in Southern Ethiopia (56.6%) [9], however, it is lower than the 12 months discontinuation rate of 77.6% in another study [1]. The discrepancy could be due to the difference in the composition of study participants, as almost all the study participants in our study lived in urban areas.

However, the current discontinuation prevalence is higher compared with studies conducted in Yemen (47%), Cambodia (43%) [19], Mekele City (38%) [16], and from the 2016 Ethiopian Demographic and Health Survey (21.6%) [13]. A possible explanation for the difference is young clients in this this study may prefer the implant to space pregnancy for a shorter period of time.

In this study, the likelihood of early discontinuation of LARC among women who experienced side effects was 1.9 times higher than those who did not report those side effects, similar to other studies [5, 11, 16]. This could be because, contrary to what many people expect, LARC devices can cause side effects, so if women are choosing them hoping for fewer side effects, they are likely to be disappointed. This is comparable to other studies from Butajira, Nigeria and United Kingdom [20–22]. This is an indication of clients’ intolerance for clinically minor side effects, particularly in the absence of high-quality counseling. Previous work in Ghana has demonstrated that family planning counselors do not discuss potential side effects [23]. Changes in the menstrual cycle, especially during early period of insertion due to the method might make women remove their device prematurely. Ensuring women are accepting devices which cause side effects which are tolerable to them is an important part of the counseling session. Women need to be presented with the possible side effects so they can choose a method which meets their preferences, both for efficacy and for side effect profile.

This study showed that women who reported they were not counseled about the side effects of LARC before insertion were 3.5 times more likely to discontinue early, which supports findings from other studies [11, 15, 17, 24]. Women who are adequately counseled on possible side effects will be less surprised by them and know how to manage them with them.

There is 3.5 times increased risk for early discontinuation of LARC if the women have history of abortion compared with no abortion history. This is consistent with two previous studies [5, 25].

Participants who had not utilized contraception had a 2.9-fold increase in early discontinuation compared with women who have used modern contraception before this LARC. In the other studies, previous contraceptive use has had no significant association with early discontinuation. In the present study, this might be due to tolerance of side effects, better understanding about LARC and the importance of overall family planning. This could also be due to women with no previous experience trying a method for the first time and not being satisfied. Women who have used other methods previously might be more satisfied with this LARC device after trying different methods previously.

Evidence from the 2016 Ethiopian Demographic and Health Survey [18] showed that family planning service providers may pressure women to accept long-acting methods rather than short-acting methods which had increased discontinuation rate. The current study demonstrated that clients who choose LARC by themselves were significantly less likely to discontinue early compared with those influenced by others. Even though not currently in practice, previously, the Government had tried to increasing the share of LARC in overall contraceptive use and health care providers were evaluated based on the number of LARC devices placed. This might have biased providers to push clients to choose LARC despite their interest, and subsequently clients discontinue that method because it was not what they wanted.

Clients who had their LARC insertion at SPHHMC were less likely to discontinue early compared with other health facility insertion. This could be because at SPHMMC there are family planning subspecialists, fellows, and residents, who are involved in patient counseling about possible side effects and benefits that enable clients to clearly decide their own choice. Additionally, the initiation of family planning counseling during ANC follow-up and provision during immediate post-partum period might increase clients’ satisfaction which minimizes risk of early discontinuation.

The strength of this study is that all previous studies of LARC discontinuation in Ethiopia used a community-based approach, with varying study period [1, 16, 17]. This study provided information from four big hospitals in urban Ethiopia.

This study is not without limitations. Firstly, it was conducted at urban hospitals, while most of the Ethiopia population resides in rural settings. Thus, the generalizability is limited. Further, some clients likely have recall bias on the date of their LARC insertion. Finally, quality of counseling was not assessed objectively and so it is hard to determine if patients were presented with side effects or not during counseling.

In conclusion the overall prevalence early discontinuation of long-acting reversible contraceptive was high among study participants. Lack of pre-insertion counseling on side effects, experiencing side effects, history of abortion, no prior contraception use, insertion in a facility other than SPHMMC, and influence on choice of contraceptives by others are significantly associated with early discontinuation.

Providing in-depth counseling about possible side effects and giving an opportunity. for the clients to decide their own choice of contraception might reduce early discontinuation rate.

## Data Availability

We will present data and materials immediately upon request.
